# Dynamic changes in the glycocalyx and clinical outcomes in patients undergoing endovascular treatments for large vessel occlusion

**DOI:** 10.3389/fneur.2023.1046915

**Published:** 2023-01-26

**Authors:** Dan Liang, Xiuli Zeng, Mingzheng Yao, Fei Li, Jiaxing Lin, Liang Zhang, Jialin Liu, Li'an Huang

**Affiliations:** ^1^Department of Neurology, The First Affiliated Hospital, Jinan University, Guangzhou, China; ^2^Department of Neurology, The Sixth Affiliated Hospital, School of Medicine, South China University of Technology, Foshan, China; ^3^Department of Neurology, Meizhou People's Hospital, Meizhou, China

**Keywords:** large vessel occlusion, endovascular therapy, ischemia/reperfusion injury, glycocalyx, outcomes

## Abstract

**Purpose:**

We aimed to verify the prognostic value of the glycocalyx as a marker of blood–brain barrier damage in patients with acute ischemic stroke undergoing endovascular therapy.

**Methods:**

We recruited patients with large vessel occlusion who were undergoing recanalization and tested their glycocalyx at multiple time points. On the basis of the 90-day follow-up data, the patients were divided into a survivor group and a nonsurvivor group. In addition, neurological function was tracked, and patients were divided into a neurological deterioration group and a group without neurological deterioration. Associations between outcomes and dynamic changes in the glycocalyx were determined using a linear mixed model, and significant factors were used as covariates.

**Results:**

Nonsurvivors and patients with neurological deterioration had significantly higher syndecan-1 concentrations than survivors and patients without neurological deterioration, and syndecan-1 tended to decline after endovascular therapy (*p* < 0.05). The increased level of syndecan-1 at 36 h after endovascular treatment was positively correlated with the National Institute of Health Stroke Scale score for neurological deterioration (*r* = 0.702, *p* = 0.005). However, there was no significant difference in the level of hyaluronic acid or heparan sulfate in the plasma of patients with different clinical outcomes.

**Conclusion:**

Pre-reperfusion syndecan-1 levels in patients with large vessel occlusion stroke are associated with 90-day mortality and the re-degradation of syndecan-1 is positively associated with neurological deterioration.

## 1. Introduction

Despite advances in reperfusion strategies, ischemic stroke remains a significant cause of death and disability; these effects are, to a certain degree, due to dysfunction caused by symptomatic intracranial hemorrhage and malignant cerebral edema (1). In the past, it was believed that under ischemic stroke conditions, the destruction of the blood–brain barrier (BBB) tight junction integrity directly led to angioedema, hemorrhagic transformation, and increased mortality (2). With further research, however, investigators found that it was the glycocalyx rather than the tight junctions that caused vessel leakage, as the tight junctions remained intact while BBB leakage was observed (3, 4).

The glycocalyx, which covers the luminal surface of endothelial cells, is composed of glycoproteins containing acidic oligosaccharides and terminal sialic acid, proteoglycan, and glycosaminoglycan side chains, primarily hyaluronic acid (HA), syndecan-1 (SDC-1), and heparan sulfate (HS) (5). The glycocalyx forms a protective surface layer for blood vessels, but this coating is vulnerable to damage, for example, from diabetes (6), sepsis (7), and ischemia/reperfusion injury (8, 9). Degradation of the glycocalyx can affect the integrity of the BBB (10). Elevated blood levels of glycocalyx components may be a sign of glycocalyx degradation (11). Recent studies have identified glycocalyx components as biomarkers of ischemic stroke (12–14), and they are associated with neurological deterioration (ND) as well as increased mortality (11). In this study, we sought to determine the dynamic change in the glycocalyx after the recanalization of blood vessels through longitudinal monitoring and to unravel the potential correlation of the glycocalyx with clinical outcomes.

## 2. Methods

### 2.1. Study design and population

A total of 101 adult patients with large vessel occlusion (LVO) were consecutively admitted to the First Affiliated Hospital of Jinan University within 24 h after stroke onset from November 2019 to January 2021. Among these patients, 21 were excluded due to blood sample hemolysis and loss of follow-up. Eighty patients were included in the final analysis. The study was approved by the Medical Ethics Committee of the First Affiliated Hospital of Jinan University (No. KY-2020-104).

Patients who met the following criteria were enrolled in the study: diagnosis of acute ischemic stroke, absence of large vessels on brain computed tomography angiography, receipt of endovascular therapy [modified thrombolysis in cerebral infarction score 2b/3 (15)], and willingness to provide informed consent. The exclusion criteria were as follows: diagnosis of hemorrhagic stroke, major trauma, acute infectious disease, autoimmune disease, severe hypoproteinemia, cardiogenic shock, or cancer. The main outcome of the study was 90-day mortality, which was assessed by a modified Rankin Scale (mRS) from a telephone follow-up, and the secondary outcome was neurological deterioration (ND) within 7 days after endovascular treatment. The patients were divided into an ND group and a non-ND (nND) group according to whether neurologic deterioration occurred within 7 days after endovascular treatment. In addition, patients were divided into the non-survivor group (mRS = 6) and the survivor group (mRS < 6) according to the 90-day mRS score.

### 2.2. Clinical variables

**Figure 1:**
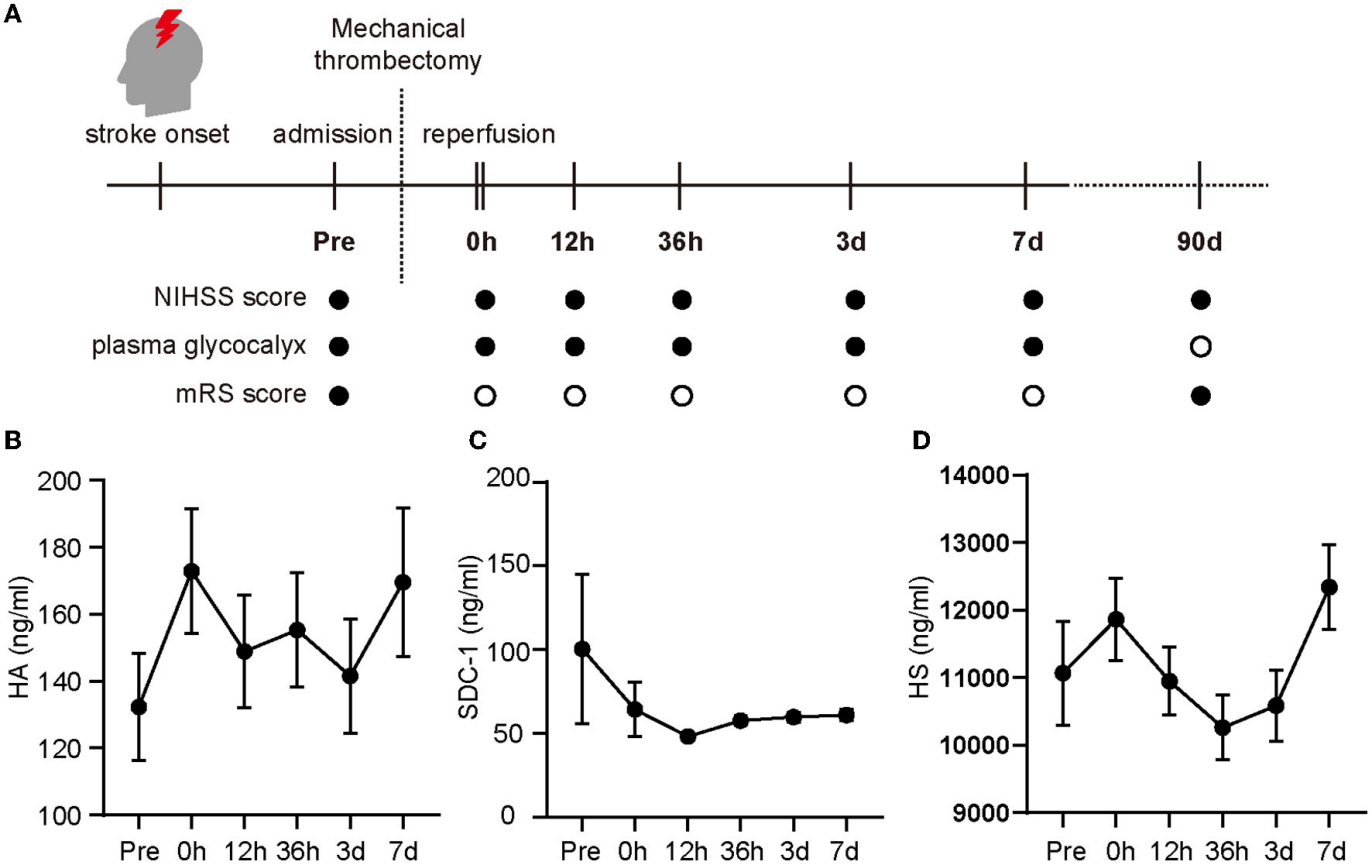
Changes in glycocalyx components in the plasma of patients enrolled in the study. The evaluation index and multiple time points (pre-operation (referred to as “Pre” in the figure), 0 h, 12 h, 36 h, 3 days, and 7 days after the operation) during the investigation are shown in **(A)** (• for evaluation, ◦ for no evaluation) and the glycocalyx concentrations in the plasma are shown from **(B–D)**. Differences between groups were compared using a linear mixed model. NIHSS, National Institute of Health Stroke Scale; mRS, modified Rankin Scale; HA, hyaluronic acid; SDC-1, syndecan-1; HS, heparan sulfate.

During hospitalization, patients were subjected to repeated blood sampling and clinical evaluation before surgery and at 0 h, 12 h, 36h, 3 days, and 7 days after endovascular therapy, and they were followed up at 90 days after onset (Figure 1A) and after obtaining the consent. Baseline demographic and clinical data were obtained in patient records, including age, sex, vascular risk factors (hypertension, hyperlipidemia, and diabetes mellitus), smoking, alcohol, and time from onset to recanalization. The modified thrombolysis in cerebral infarction (mTICI) score, scored by two professional physicians, was used to grade angiographic outcomes after endovascular thrombectomy. Additionally, stroke severity was scored on the National Institutes of Health Stroke Scale (NIHSS). ND was characterized as an increase of ≥4 points in the NIHSS score, ≥1 point in the consciousness score (1a−1c), ≥1 point in the motor score (5a−5b), or any new neurological deficits (that cannot be measured by the NIHSS score) within 7 days (16). Survivors were defined as those who were alive at the 90-day follow-up after stroke onset, and the mRS scores were obtained by a telephone follow-up.

### 2.3. Measurement of the glycocalyx

Blood samples were repeatedly drawn in tubes containing ethylenediaminetetraacetic acid at the multiple time points mentioned above. All samples were centrifuged for 10 min at 3,000 rpm, and afterward, plasma samples were stored at −80°C until further experiments. The concentrations of HA (Cloud-clone Co., Wuhan, China), SDC-1 (Cusabio Biotech Co., Wuhan, China), and HS (Cusabio Biotech Co., Wuhan, China) were quantified by enzyme-linked immunosorbent assay kits.

### 2.4. Data analysis

Categorical variables were compared using the chi-square test and are presented as percentages. Continuous variables were compared using the *t*-test or the Wilcoxon rank-sum test as appropriate. Significant factors were used as covariates for further analysis. Significant correlations were tested by Spearman's rank correlation coefficient. Differences between groups were evaluated using a linear mixed model (17). All patients were taken as subjects, and each time point measured was taken as the repeated index. The repeat covariance type was selected as AR (1), while HA, SDC-1, and HS were used as the dependent variables, and the repeat times and groups were used as fixed effect factors to compare the main effects, with confidence intervals adjusted for the Bonferroni correction. The significance threshold was set to 0.05. Statistical analyses were performed using SPSS Statistics Version 27.0 (IBM Corporation; Armonk, NY, USA).

## 3. Results

### 3.1. Clinical characteristics of the participants

Old age and history of coronary heart disease were more common in non-survivors than survivors (age: *p* = 0.015; history of coronary heart disease: *p* = 0.017). Patients who received intravenous thrombolysis treatment tended to have lower mortality than those who did not receive this treatment (*p* = 0.035, Table 1). In addition, female sex and coronary artery disease were more common in patients with ND (sex: *p* = 0.004; coronary artery disease: *p* = 0.030), and patients with atrial fibrillation were more prone to ND as well (*p* = 0.0042, Table 2).

**Table 1:** Clinical Baseline Characteristics in survivors and death.

	**All** **(*n =* 80)**	**Survivors** **(*n =* 68)**	**Nonsurvivors** **(*n =* 12)**	***p*-value**
**Clinical items**				
Age (years)	67.91	66.40 ± 13.33	76.50 ± 10.54	0.015^*^
Sex (male; *n* %)	48 (60)	43 (89.58)	5 (10.42)	0.160
Time of reperfusion (h)	8.26 (5–10.75)	8.43 ± 5.18	7.33 ± 4.08	0.491
Basal NIHSS	16 (12-20)	15.65 ± 5.24	18.50 ± 6.76	0.101
Intravenous thrombolysis (*n* %)	32 (40)	31 (96.88)	1 (3.12)	0.035^*^
**Medical history**				
Hypertension (*n* %)	52 (65)	43 (82.69)	9 (17.31)	0.646
Diabetes mellitus (*n* %)	26 (32.5)	20 (76.92)	6 (23.08)	0.160
Hyperlipide (*n* %)	27 (33.8)	23 (85.2)	4 (14.8)	1.000
Coronary artery disease (*n* %)	14 (17.5)	9 (64.29)	5 (35.71)	0.017^*^
Atrial fibrillation (*n* %)	39(47.5)	31 (79.49)	8 (20.51)	0.301
WBC, 10^∧^9/L	10.21 ± 4.01	10.11 ± 3.46	10.76 ± 6.49	0.742
Neu, 10^∧^9/L	8.18 ± 4.02	8.08 ± 3.63	8.75 ± 5.94	0.597
TC, mmol/L	4.48 ± 1.22	4.55 ± 1.20	4.01 ± 1.30	0.217
TG, mmol/L	1.36 ± 1.55	1.39 ± 1.65	1.22 ± 0.39	0.764
HDL, mmol/L	0.97 ± 0.22	0.98 ± 0.22	0.92 ± 0.27	0.468
LDL, mmol/L	2.74 ± 0.90	2.80 ± 0.87	2.34 ± 1.04	0.149
CRP, mg/L	30.87 ± 38.57	31.74 ± 39.08	25.94 ± 36.71	0.634

Categorical variables were compared using the chi-square test, and continuous variables were compared using the t-test or Wilcoxon rank-sum test as appropriate. Numbers are the mean (SD), median (IQR), or frequency (percentage). ^*^p < 0.05. SD, standard deviation; IQR, interquartile range; NIHSS, National Institute of Health Stroke Scale; WBC, white blood cell; Neu, neutrophil; TC, total cholesterol; TG, triglyceride; HDL, high-density lipoprotein; LDL, low- density lipoprotein; CRP, C-reactive protein.

**Table 2:** Clinical Baseline Characteristics in ND and nND.

	**All**	**nND** **(*n =* 63)**	**ND** **(*n =* 17)**	***p*-value**
**Clinical items**				
Age (years)	67.91	66.54 ± 13.65	73.00 ± 11.34	0.077
Sex (male; *n* %)	48 (60)	43 (89.6)	5 (10.4)	0.004^*^
Time of reperfusion (h)	8.26 (5–10.75)	8.71 ± 5.31	6.62 ± 3.41	0.129
Basal NIHSS	16 (12–20)	15.61 ± 5.11	17.76 ± 6.84	0.158
Intravenous thrombolysis (*n* %)	32 (40)	28 (87.5)	4 (12.5)	0.118
**Medical history**				
Hypertension (*n* %)	52 (65)	38 (73.1)	14 (26.9)	0.091
Diabetes mellitus (*n* %)	26 (32.5)	18 (69.2)	8 (30.8)	0.149
Hyperlipide (*n* %)	27 (33.8)	24 (88.9)	3 (11.1)	0.114
Coronary artery disease (*n* %)	14 (17.5)	8 (57.1)	6 (42.9)	0.030^*^
Atrial fibrillation (*n* %)	39(47.5)	27 (69.2)	12 (30.8)	0.042^*^
WBC, 10^∧^9/L	10.21 ± 4.01	10.02 ± 3.53	10.90 ± 5.51	0.427
Neu, 10^∧^9/L	8.18 ± 4.02	7.97 ± 3.69	8.95 ± 5.13	0.375
TC, mmol/L	4.48 ± 1.22	4.57 ± 1.23	4.05 ± 1.08	0.157
TG, mmol/L	1.36 ± 1.55	1.42 ± 1.70	1.12 ± 0.33	0.530
HDL, mmol/L	0.97 ± 0.22	0.97 ± 0.22	0.98 ± 0.25	0.816
LDL, mmol/L	2.74 ± 0.90	2.83 ± 0.89	2.35 ± 0.87	0.091
CRP, mg/L	30.87 ± 38.57	30.70 ± 38.47	31.48 ± 40.10	0.941

Categorical variables were compared using the chi-square test and continuous variables were compared using the t-test or Wilcoxon rank-sum test as appropriate. Numbers are the mean (SD), median (IQR), or frequency (percentage). ^*^p < 0.05. ND, neurological deterioration; nND, non-neurological deterioration; SD, standard deviation; IQR, interquartile range; NIHSS, National Institute of Health Stroke Scale; WBC, white blood cell; Neu, neutrophil; TC, total cholesterol; TG, triglyceride; HDL, high-density lipoprotein; LDL, low- density lipoprotein; CRP, C-reactive protein.

### 3.2. Dynamic changes in the glycocalyx in patients with LVO

After cerebral arterial occlusion, the concentrations of HA, SDC-1, and HS in the plasma were 132.29 ± 16.06, 100.39 ± 44.67, and 1,1066.70 ± 767.82 ng/ml, respectively. HA (Figure 1B) and HS concentrations (Figure 1D) continued to rise immediately after recanalization (0 h) and then fluctuated. In contrast, the SDC-1 level dropped rapidly after reperfusion and peaked again 36 h later (Figure 1C).

### 3.3. Concentrations of glycocalyx components and ND

**Figure 2:**
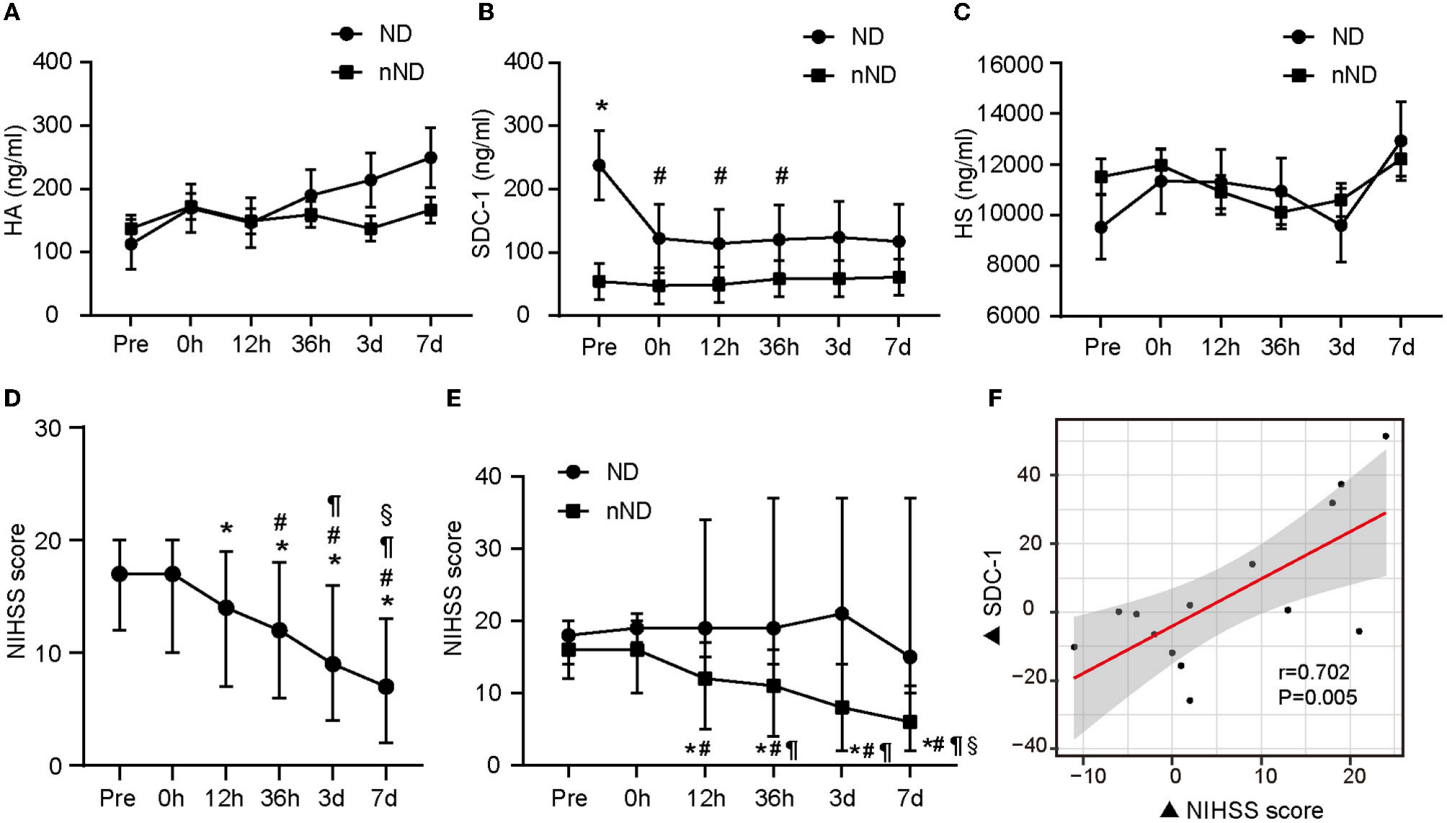
The glycocalyx components of different evolution and the relationship between glycocalyx and neurological function. The difference in glycocalyx levels between the ND group and the nND group is shown from **(A–C)**. Neurologic changes in all patients included in the study are presented in **(D)**. The differences in NIHSS scores between ND and nND groups are shown in **(E)**. The relationship between the change of NIHSS score and the change of SDC-1 level (based on the level at admission) at 36 h after stroke onset is shown in **(F)**. A linear mixed model was used to compare component concentrations between groups. *p < 0.05 vs. nND and ^#^*p* < 0.05 vs. pre-operation in **(B)** respectively. *represents *p* < 0.05 vs. pre-operation, ^#^represents *p* < 0.05 vs. 0 h after the operation, ^¶^represents *p* < 0.05 vs. 12 h after the operation, ^§^represents *p* < 0.05 vs. 36 h after the operation in **(D, E)**. Significant correlations were tested by Spearman's rank correlation coefficient. Pre, pre-operation; 0 h, 0 h after the operation; 12 h, 12 h after the operation; 36 h, 36 h after the operation; 3 days, 3 days after the operation; 7 days, 7 days after the operation; ND, neurological deterioration; nND, non-neurological deterioration; NIHSS, National Institute of Health Stroke Scale; HA, hyaluronic acid; SDC-1, syndecan-1; HS, heparan sulfate.

Patients who developed ND showed higher SDC-1 levels than patients without ND (Figure 2B, *n* = 17, *p* = 0.004). Compared with preoperative levels, patients' postoperative SDC-1 levels were significantly decreased (0 h: *p* = 0.00003, 12 h: *p* = 0.002, and 36 h: *p* = 0.045). The plasma HA level of patients with ND gradually increased, while that of the patients without ND fluctuated but did not significantly change within 7 days after onset, but the difference between groups was not statistically significant (Figure 2A, *p* > 0.05). Changes in HS among ND and nND patients were almost synchronized (Figure 2C, *p* > 0.05).

After endovascular therapy, the neurological function of the evaluated patients overall was significantly improved (shown in Figure 2D). However, the NIHSS score increased again in the ND group from 36 h to 3 days after endovascular therapy (Figure 2E). We found that plasma SDC-1 levels were also increased in these patients. Subsequently, we detected that ΔNIHSS at 36 h after reperfusion (the increase in the NIHSS score since admission) was positively correlated with ΔSDC-1 (the increase in SDC-1 since admission) at the same time point in patients in the ND group (*n* = 17, *r* = 0.702, *p* = 0.005) (shown in Figure 2F).

### 3.4. Glycocalyx concentration and outcomes

**Figure 3:**
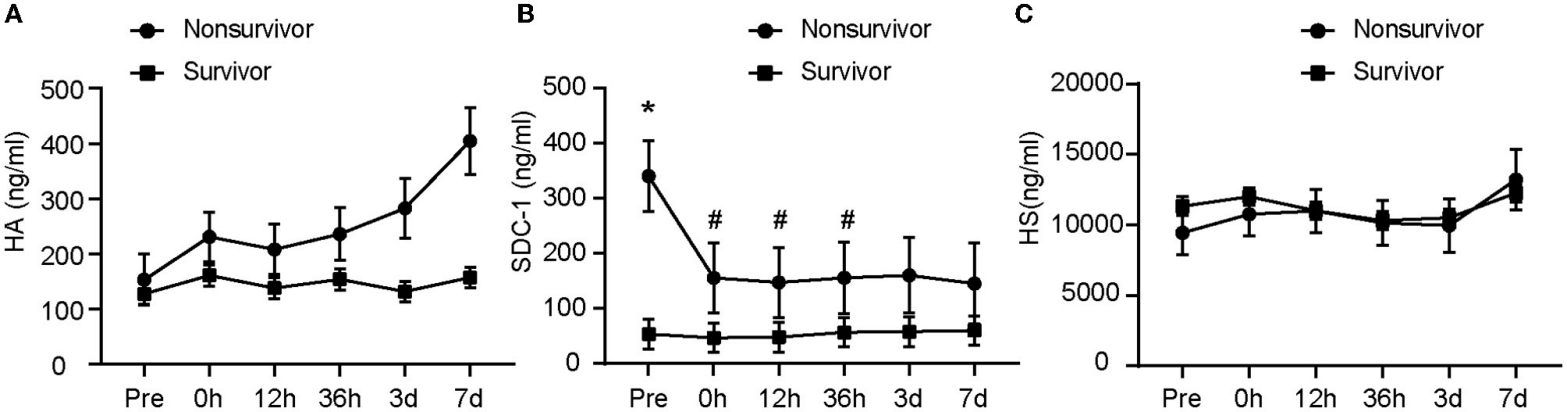
The glycocalyx components at different outcomes. The difference in glycocalyx levels between the nonsurvivor group and the survivor group is shown from **(A–C)**. *represents *p* < 0.05 vs. survivor; ^#^represents *p* < 0.05 vs. Pre. A linear mixed model was used to compare component concentrations between groups. Pre, pre-operation; 0 h, 0 h after the operation; 12 h, 12 h after the operation; 36 h, 36 h after the operation; 3 days, 3 days after the operation; 7 days, 7 days after the operation; HA, hyaluronic acid; SDC-1, syndecan-1; HS, heparan sulfate.

Our data showed a significant association among SDC-1 concentration, time, and 90-day survival, as detected by linear mixed models (Figure 3). Data analysis revealed a significantly higher preoperative concentration of SDC-1 in nonsurvivors (*n* = 12) than in survivors (*n* = 68) (*p* < 0.001). Immediately after endovascular treatment, plasma SDC-1 levels in nonsurvivors decreased significantly (*p* < 0.001), whereas plasma SDC-1 levels in survivors showed little fluctuation (Figure 3B). Similarly, there was no significant change in HA in the plasma of the survivors within 7 days of onset, whereas the plasma concentration of HA in the non-survivors gradually increased (Figure 3A). The changes in plasma HS were almost identical between the two groups. The difference between HA and HS was not statistically significant (Figure 3C).

## 4. Discussion

Although endovascular therapy can successfully rescue ischemic brain tissue, stroke remains the second leading cause of death worldwide. Brain edema and hemorrhage transformation are the main complications that affect rehabilitation after reperfusion therapy (1), but there is no effective prediction method at present. The destruction of the BBB is one of the main pathological mechanisms of cerebral edema and hemorrhagic transformation and has received a lot of attention. In recent years, considerable efforts have been devoted to exploring the relationship between biomarkers and stroke. The glycocalyx is the main component that maintains the function of the BBB, and the levels of glycocalyx molecules are elevated in the plasma of patients with stroke. In this study, we investigated the dynamic change of the glycocalyx in patients who have received endovascular therapy for LVO and analyzed the relationship between glycocalyx and clinical prognosis.

Studies have shown that women's general health status, cerebrovascular anatomy, and function have important effects on ischemic stroke, making the prognosis for acute ischemic stroke worse in women than in men (18, 19). The differences between men's and women's results in the present study are consistent with recent major studies on prognostic risk factors for acute ischemic stroke. In addition, coronary artery disease and atherosclerosis make the polysaccharide envelope on the vessel wall thinner, making it less resistant to ischemic and hypoxic damage (20). Conversely, the glycocalyx in patients with atrial fibrillation tends to be relatively intact and their symptoms are milder when the glycocalyx is impaired by some other factor.

Previous studies have shown that increasing levels of glycocalyx components can be detected in the plasma of patients after stroke onset. Nevertheless, no significant changes in SDC-1 were observed, while HA and HS increased significantly after onset (12, 14). To our knowledge, this is the first study to describe the dynamic changes in the plasma concentrations of glycocalyx components in patients with LVO and to analyze glycocalyx shedding in blood samples collected before endovascular treatment. We found that SDC-1 increased significantly in the early stage of stroke and decreased rapidly after reperfusion therapy, and the peak concentrations of HA and HS appeared later, suggesting that SDC-1 is more sensitive to ischemia/reperfusion injury.

Ischemia-induced glycocalyx injury occurs early and is a strong trigger for BBB dysfunction. We found that the plasma concentration of SDC-1 was elevated in patients with ND and increased synchronously with the degree of deterioration. SDC-1 significantly correlated with BBB leakage. Degradation of the glycocalyx results in the shedding of SDC-1 and phosphorylation of its cytoplasmic domain (4). Phosphorylation of SDC-1 can recruit cortical proteins, thereby promoting efficient actin-dependent endocytosis and increasing the permeability of the BBB (21). Furthermore, the shedding of SDC-1, the molecule that serves as the last safeguard of the BBB, increases the interaction of blood components with endothelial cells, allowing macromolecules to pass through the barrier (5). Damage to the BBB destabilizes the internal environment of the central nervous system, thus preventing the maintenance of normal neurological function. We found that fluctuations in plasma SDC-1 concentration were more pronounced in patients with LVO than in patients with mild neurological deficits (12), consistent with animal experiments (11).

Our data showed a biphasic peak of SDC-1 concentration, synchronizing with the biphasic pattern of the BBB condition (22). Notably, the second stage of the post-reperfusion change in BBB permeability occurs 18 to 96 h after reperfusion and is characterized by leukocyte infiltration (22). Inflammation plays an important role in the development of ischemia/reperfusion injury (23–25). In SDC-1 gene-deficient rats, the interaction between leukocytes and endothelial cells is enhanced. SDC-1 promotes the recruitment of neutrophils by combining with chemokines released in the degradation process to increase the inflammatory response, leading to endothelial dysfunction and damage to the BBB (26). A recent study showed that glucocorticoid administration reduced the inflammatory response caused by ischemia/reperfusion injury, decreased the permeability of the BBB, and improved mortality and neurological function in a cardiac arrest/cardiopulmonary resuscitation rat model (11).

These results suggested that severe ischemic and hypoxia after LVO leads to the shedding of the glycocalyx, which weakens the BBB physical barrier and exacerbates the BBB damage through inflammatory reaction, thus increasing the occurrence of symptomatic intracranial hemorrhage and brain edema. These prior reports are consistent with our findings that patients with a greater degree of ND or death consistently exhibited higher levels of SDC-1. SDC-1 is also an extremely early predictor of survival before endovascular therapy and may therefore be useful as both an early biomarker of clinical outcome and a therapeutic target to increase survival. Overall, our study showed an association between endothelial shedding and ND, as well as adverse outcomes.

## 5. Limitations

Our study has many limitations. The first and most important limitation of our study is the small sample size, a result of the challenging clinical environment. Second, the duration of the study was only 7 days and no observation data were obtained over a longer period. There is no further information as to when the levels of HA, SDC-1, and HS in the plasma of patients with stroke dropped to the same levels found in healthy individuals. Third, we do not have data on the concentrations of the glycocalyx components in patients before the onset of stroke; therefore, we could not conduct a comparison of ischemia/reperfusion damage or determine when the damage stopped.

## 6. Conclusion

In this study, we show the fluctuation of glycocalyx components in the plasma of patients with stroke after endovascular treatment and identify the association between plasma SDC-1 levels and deterioration of neurological function. Furthermore, we show that the pre-reperfusion level of SDC-1 can help predict 90-day mortality. Despite the limited number of subjects, the results of our investigation are still encouraging for further research.

## Data availability statement

The original contributions presented in the study are included in the article/Supplementary material, further inquiries can be directed to the corresponding author.

## Ethics statement

The studies involving human participants were reviewed and approved by the Medical Ethics Committee of the First Affiliated Hospital of Jinan University. The patients/participants provided their written informed consent to participate in this study.

## Author contributions

DL was involved in the design of the trial, collected, verified, analyzed the data, and wrote the first draft of the article. XZ and LH participated in the design of the trial, analyzed the data, and revised the manuscript. MY, FL, JLin, and LZ performed the data analysis. JLiu collected the blood samples. All contributors have read and approved the final manuscript.
